# Development of a novel quantitative lateral flow assay for vancomycin for therapeutic drug monitoring

**DOI:** 10.1038/s41598-025-09145-1

**Published:** 2025-07-08

**Authors:** Alaa Riezk, Vasin Vasikasin, Richard C. Wilson, Timothy M. Rawson, Anthony E. G. Cass, Alison H. Holmes

**Affiliations:** 1 Centre for Antimicrobial Optimisation, Imperial College London, London, UK; 2 NIHR Health Protection Research Unit in Healthcare Associated Infections and Antimicrobial Resistance, Hammersmith Hospital, Imperial College London,, Du Cane Road, London, UK; 3 Department of Internal Medicine, Phramongkutklao Hospital and Phramongkutklao College of Medicine, Bangkok, Thailand; 4 David Price Evans Global Health and Infectious Diseases Research Group, University of Liverpool, Liverpool, UK; 5 Fleming Initiative, Fleming Centre, Imperial College London and Imperial College Healthcare NHS Trust, London, UK

**Keywords:** Therapeutic drug monitoring, Vancomycin, Lateral flow assay, Point of care, Infectious diseases, Assay systems

## Abstract

Antibiotic optimisation through therapeutic drug monitoring (TDM) is a key strategy in tackling antimicrobial resistance. However, current quantification methods rely on laboratory-based equipment, delaying timely dose adjustments. We developed a novel point-of-care lateral flow assay (LFA) for the quantification of vancomycin in serum. Unlike conventional LFAs with a single test and control line, this assay incorporates two test lines: one for vancomycin binding and one for avidin capture of excess conjugate. Gold nanoparticles were conjugated to biotinylated bovine serum albumin–vancomycin, enabling competitive binding with anti-vancomycin antibodies and avidin capture. Signal ratios from smartphone-acquired images were quantified using an automated image analysis algorithm. Applied to vancomycin in serum, the LFA demonstrated a quantitative detection range of 2.88 to 45,000 ng/mL, with good accuracy, reproducibility, and high recovery in spiked samples. This approach offers a promising solution for decentralised, rapid TDM in clinical settings, especially where timely dose optimisation is critical.

**Supplementary Information:**

The online version contains supplementary material available at 10.1038/s41598-025-09145-1.

## Introduction

Antimicrobial resistance (AMR) poses a significant global threat to human health and well-being^1^. One critical strategy in addressing AMR is antibiotic dose optimisation through therapeutic drug monitoring (TDM), which involves measurement of plasma antibiotic concentrations to guide dose adjustments^2^. Standard guidelines emphasise the importance of TDM for vancomycin, as it reduces the risk of nephrotoxicity and ensures therapeutic efficacy^3^. Vancomycin is commonly used to treat serious Gram-positive infections, including methicillin-resistant Staphylococcus aureus (MRSA), and maintaining appropriate therapeutic levels is essential for clinical success^4^. However, the prolonged turnaround times associated with conventional TDM methods hinder timely dose adjustments, limiting their clinical impact^5^.

Commercially available assays for vancomycin, including liquid chromatography/mass spectrometry (LC/MS), enzyme-linked immunosorbent assays (ELISA), and fluorescence-based methods, are often centralised and labour-intensive^6^. These techniques typically require sample transportation to a laboratory^7^, resulting in a turnaround time of 2–3 h, with actual dose adjustments occurring ≥ 12 h after blood collection. Despite advancements, improvements in simplicity, speed, and cost-effectiveness remain limited^7^. There remains an unmet need for decentralised, rapid, and user-friendly approaches to support timely vancomycin dose optimisation at the point of care.

The development of fast and easy-to-use point-of-care devices for vancomycin TDM offers a solution to these challenges. Lateral flow assays (LFA) have emerged as a promising technology that aligns with the World Health Organization (WHO) ASSURED criteria for diagnostics: affordable, sensitive, specific, user-friendly, rapid, robust, equipment-free, and deliverable to end-users^8,9^. Traditionally used for qualitative analysis, recent advances in affordable detection devices, including smartphone cameras, have enabled quantitative applications of LFAs for antibiotic detection^10^.

An LFA typically consists of a nitrocellulose membrane with immobilised biological molecules, employing either a sandwich or competitive format. For small molecules like antibiotics, a competitive LFA format is necessary^8,11,12^. While competitive LFAs have demonstrated potential for providing rapid, quantitative results at the POC^6,12,13^, traditional designs face limitations, including narrow dynamic ranges and reliance on specialised detectors for sensitivity. For vancomycin, these limitations hinder accurate quantification at clinically relevant concentrations (15–20 mg/L), necessitating either extensive dilution or the use of controlled laboratory conditions, which undermines their utility at the point of care^14^.

To overcome these limitations, we developed a novel competitive LFA format featuring two test lines: an antibody line and an avidin line. This design leverages biotin-avidin interactions to capture excess conjugate, enabling differential signal analysis from the two lines. Concentration can be extrapolated from an analysis of the intensity of both lines. This approach significantly enhances accuracy across a broader dynamic range, encompassing clinically relevant antibiotic concentrations.

The study was conducted in two phases. In the first phase, a novel competitive LFA was developed and optimised for the quantification of vancomycin in serum samples. The performance of this LFA was compared to established methods, including ELISA and a traditional competitive LFA for vancomycin. In the second phase, the optimised LFA was further validated using spiked serum samples to assess its accuracy, reproducibility, specificity, and practical applicability in therapeutic drug monitoring.

## Materials and methods

### Chemicals and reagents

Vancomycin hydrochloride, bovine serum albumin (BSA), 1-Ethyl-3-[3-dimethylaminopropyl] carbodiimide hydrochloride (EDC), gold nanoparticles (40 nm diameter) in 0.1 mM phosphate-buffered saline (PBS) buffer, PBS, polyethylene glycol (PEG) 6,000 Da, avidin, Anhydrous Na_2_CO_3_, anhydrous NaHCO_3_, H_2_SO_4,_ phosphate buffered saline (PBS) tablets, Tween^®^-20, membrane cut-off value 12 KDa, Pur-A-Lyzer™ Mega Dialysis Kit– were purchased from Sigma-Aldrich. EZ-Link™ Sulfo-NHS-LC-Biotinylation Kit was purchased from ThermoFisher Scientific (Waltham, MA). Sheep anti vancomycin IgG, donkey anti sheep/goat IgG: HRP, HRP-linked anti-his antibody, 3,3′,5,5′-tetramethylbenzidine (TMB) Core + were purchased from Bio-Rad (Hercules, CA). Water was prepared with Milli-Q water system (Millipore, Burlington, MA). HiFlow Plus ^TM^ membrane, glass fibre conjugate sheet, and cellulose fibre roll were from Merck Millipore. Other common reagents were bought from Sigma-Aldrich unless specified. Printing was conducted user Automated Lateral Flow Reagent Dispenser (ALFRD) (Claremont Bio, CA, USA) incorporated with Fusion 200 Pump (Chemyx, TX).

### BSA-vancomycin conjugate preparation

Vancomycin was coupled to BSA using EDC. BSA (70 mg) was dissolved in 15 mL PBS (pH 7.4) and 1 mL of vancomycin solution (78 mg/mL in DH_2_O) was added dropwise into the beaker containing the BSA solution. Next, 1 mL of EDC (200 mg/mL in purified H_2_O) was added dropwise into the same beaker. The reaction mixture was left stirring at 500 rpm at room temperature (18–20 ^o^C) for 1 h and then moved to a cold room, where it was kept overnight at 4 °C. The next day, the conjugate was purified by dialysis. The dialysis tube was kept spinning in a beaker filled with PBS stirring at 500 rpm, at 4 °C overnight. The PBS buffer was changed three times^15,16^. Quantification of the BSA concentration post the conjugation reaction was completed using a BCA protein assay kit (ThermoFisher). After dialysis, the vancomycin BSA conjugate (BSA-Van) was stored in aliquots at − 80 °C.

### Development of indirect competitive enzyme-linked immunosorbent assay (ELISA)

An indirect competitive ELISA was developed to characterise the BSA–Van conjugate and to evaluate the quantitative binding ability of anti-vancomycin IgG. The secondary antibody used was donkey anti-sheep/goat IgG: HRP.

Briefly, 1.5 µg of BSA–Van was coated onto 96-well microtiter plates using 50 mM carbonate/bicarbonate buffer (pH 9.6) and incubated at 4 °C for 24 h. After three washes with washing buffer (0.05% Tween-20 in PBS), wells were blocked with PBS containing 1% BSA (w/v) for 1 h at 37 °C. Following another wash, 100 µL of anti-vancomycin IgG and 100 µL of vancomycin standard solutions were added to each well and incubated for 1 h at 37 °C. Plates were washed three times before adding 100 µL of the HRP-linked secondary antibody, followed by incubation for another hour at 37 °C.

After a final wash, 100 µL of TMB substrate was added and incubated at 37 °C for 15 min in the dark. The reaction was stopped with 50 µL of 2 M sulfuric acid per well, and absorbance was measured at 450 nm using a microplate reader (FLUOstar Omega, BMG Labtech, Germany). Calibration standards were prepared by serial five-fold dilution of vancomycin in serum, yielding concentrations of 1, 5, 25, 125, 625, 3,125, 15,625, and 78,125 ng/mL.

Quality control (QC) samples at three concentration levels (low, medium, high: Qc1, Qc2, Qc3) were used to evaluate assay accuracy. Intra-day precision was assessed by testing each QC level in triplicate on the same day. Inter-day precision was determined by testing the QC levels in triplicate on four consecutive days. Accuracy was evaluated as the percentage agreement between the measured and spiked QC concentrations^15–17^.

$${\text{Accuracy }}\left( {{\text{percentage}}} \right) = \left( {{\text{measured}}/{\text{known spiked}}} \right) \times {\text{100}}.$$

The calibration curve showed a linear relationship between absorbance and vancomycin concentration. Therefore, the limit of detection (LOD) and limit of quantification (LOQ) were calculated using the standard deviation of the response (σ) and the slope of the calibration curve (s), as follows:

$${\text{LOD}} = {\text{3}}.{\text{3}} \times \sigma /{\text{s}}$$

$${\text{LOQ}} = {\text{1}}0 \times \sigma /{\text{s}}$$

### Biotinylation of the BSA-Van

A standard biotinylation procedure was followed to modify the BSA-Van conjugate with NHS-LC-biotin following the ThermoFisher protocol^18^. The product was purified by dialysis and the modification by biotin was confirmed through a HABA-avidin assay^18^. To further validate the conjugation, chip-based analysis was performed with the Agilent 2100 Bioanalyzer system following Protein 80 kit protocol^19,20^. The conjugates were compared with non-biotinylated conjugates and a BSA standard under non-denaturing conditions. Finally, the biotinylated BSA-Van was stored at -80 °C for further experiments.

### Labelling anti-vancomycin IgG and conjugates with gold nanoparticles (AuNP)

AuNP labelling was performed by adding 100 µL of vancomycin IgG and 100 µL of biotinylated BSA-Van to separate 1 mL aliquots of gold nanoparticles in individual Eppendorf tubes. The pH of each AuNP solution was adjusted to 8.4 using 0.5 M NaOH. Tubes were vortexed for 5 s and placed on a shaker for 1 h at room temperature (RT). Subsequently, 25 µL of 10% BSA solution was added to each tube and incubated at RT for 3 min. The conjugates were then centrifuged at 13,500 rpm for 10 min.

After discarding the supernatant, the pellet was resuspended in 125 µL of running buffer. The running buffer used for vancomycin assays consisted of PBS containing 0.5% (v/v) Tween 20, 0.5% (v/v) Triton X-100, 0.5% (v/v) glycerol, 1% (w/v) sucrose, and 0.25% (w/v) BSA. The labelled conjugates were stored at 4 °C until use^21^.

### LFA strip assembly

**Fig. 1:**
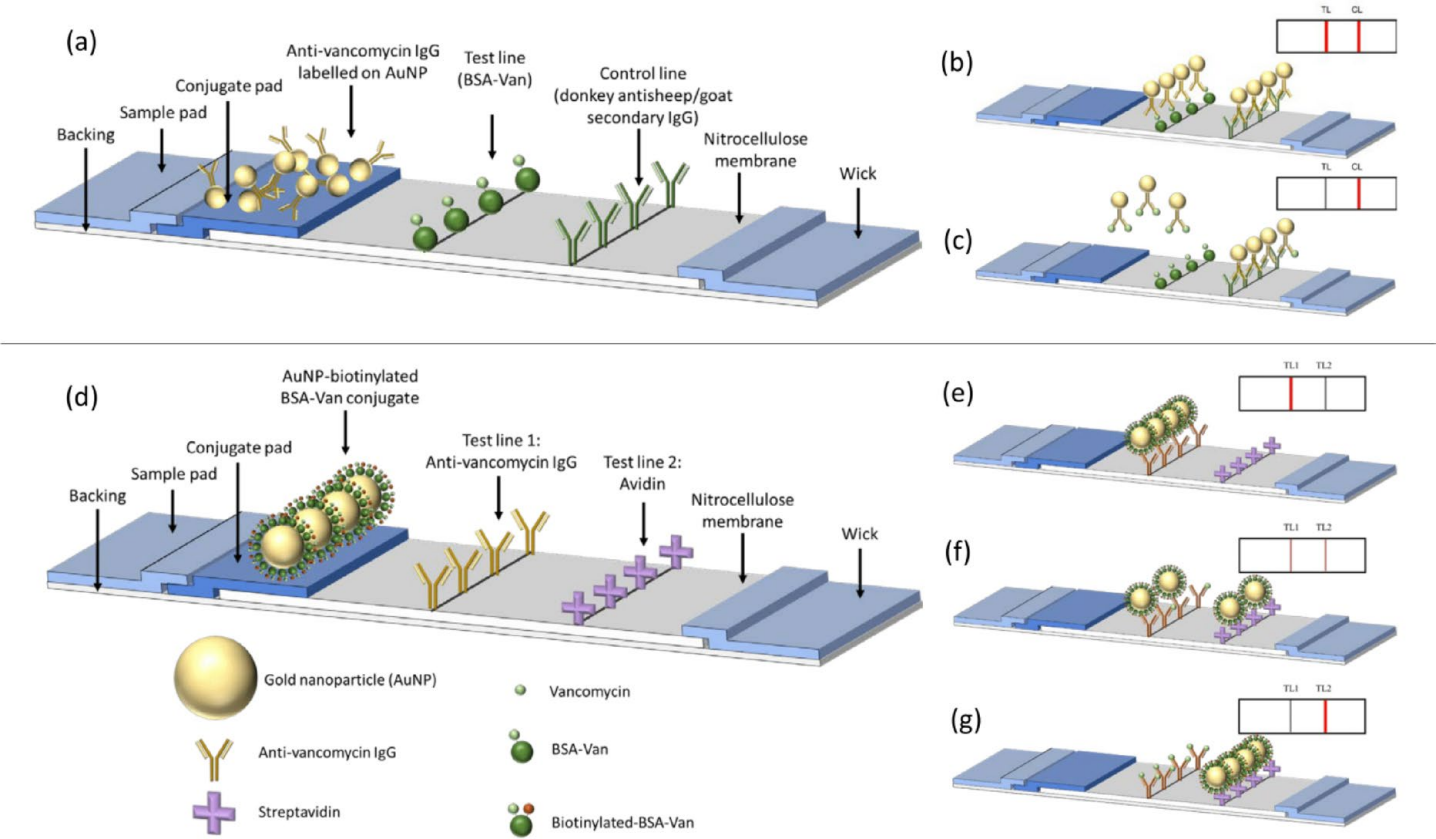
Schematic of the structure of competitive LFA (**a**–**c**) and the novel competitive LFA (**d**–**f**). (**a**) The detection antibody can bind to both antibodies on test line (TL) and control line (CL). (**b**) Negative result. (**c**) Positive result. (**d**) The detection antigen conjugated on AuNP can bind to antibodies on test line 1 (TL1) whereas the biotin can bind to test line 2 (TL2). (**e**–**g**) As analyte concentration increases, signal at TL1 decreases while signal at TL2 increases.

A traditional competitive LFA of vancomycin was developed and compared with the novel competitive LFA for vancomycin. Figure 1 represents the structure of both traditional competitive and novel competitive LFA.

For the traditional competitive LFA, vancomycin detection was based on competition between free vancomycin in the serum sample and the immobilised BSA–Van conjugate printed on the test line, both competing to bind to the anti-vancomycin IgG conjugated to gold nanoparticles. Upon application, vancomycin in the sample binds to the AuNP–IgG, forming immune complexes. Unbound AuNP–IgG is then captured by the BSA–Van on the test line. The resulting test line intensity is inversely proportional to the vancomycin concentration in the sample. Calibration standards for the traditional competitive LFA were prepared in vancomycin-free serum at concentrations of 0.12, 0.37, 1.11, 3.33, and 10 ng/mL.

For the novel competitive LFA, instead of using a traditional single test line and control line, this design incorporates two distinct test lines: one with anti-vancomycin IgG and another with avidin. Gold nanoparticles were conjugated to biotinylated BSA–Van. In the presence of vancomycin in the sample, the analyte competes with the conjugated vancomycin to bind the anti-vancomycin IgG on the first test line. Excess conjugate is then captured on the second test line via biotin–avidin binding. As the vancomycin concentration increases, the intensity of the first test line decreases, while the intensity of the second test line increases.

Conjugate pads were pre-treated with the optimised buffer alone for 24 h, followed by another 4-hour pre-treatment with the AuNP conjugates. The optimised buffer for vancomycin was the same as the running buffer. The final volume of AuNP conjugate dispensed was 30 µl per cm^2^ of the conjugate pad. The membrane backing card (7 × 30 cm) and the NC membrane (3.2 × 25 cm) were cut with a guillotine cutter and assembled with double-sided tape. The reagents [anti-Van antibody (1 mg/mL) or BSA-Van (1 mg/mL) or avidin (1 mg/mL) depending on the LFA method; Fig. 1] were taken into 1 mL syringes and dispensed on the nitrocellulose membrane using the ALFRD dispenser. The infusion rate of the syringes was 0.4 ml/min and the printing speed was adjusted to 32.5 mm/sec, allowing the volume of 0.205 µl/mm printed. The membrane with the printed test lines was left to dry at room temperature for 20 min before proceeding with strip assembly. The pre-treated conjugate pad and absorbent pad were assembled with double-sided tape onto the top and bottom of the strip, respectively. The 30 cm long NC membrane was cut into 5 mm strips, the standard width of commercial LFAs^21^.

### Rationale for dual test-line configuration

Importantly, the novel LFA format intentionally replaces the conventional test/control configuration with two functional test lines: T1 and T2. The first line (T1) is coated with anti-vancomycin antibodies to capture analyte-bound conjugate, while the second line (T2) is coated with avidin to bind excess unbound biotinylated conjugate. Although T2 primarily serves a quantitative role, its consistent presence in blank and low-concentration samples offers an indirect verification of sample migration. The role of the traditional control line is to confirm test validity when no analyte is present; however, in our design, either or both test lines must be visible for the assay to be valid, rendering an independent control line unnecessary. Future versions may incorporate a dedicated control line with a distinct nanoparticle label to further enhance robustness.

### LFA testing and readout

The assay was run with samples in optimised running buffer. A sample volume of 100 µL was used for vancomycin. Typically, the time between sample application and image capture was 5 min. The images were taken with a smartphone (Samsung A21s). After which, the images were analysed using R software to measure the intensity of the lines. The R code written for LFA reading is available at: https://github.com/vvasikasin/LFA_CAMO. Specifically, the analyses and relevant packages were implemented through a script within RStudio (PBC, Boston, MA). The core packages employed in these analyses comprised dplyr, hyperSpec, stats, baseline, and the Bioconductor package EBImage^22^. Briefly, the intensities of the test line 1 and test line 2 were normalised to the intensity of the background. The linear model was based on the concentration and the ratio of the relative intensity of both test lines to the background. The detail of this algorithm is described in Supplementary Appendix 1. The volume of sample applied to the LFA was optimised to achieve adequate membrane flow and signal development. Vancomycin required a larger volume (100 µL), likely due to its molecular size and lower permeability. This volume was selected empirically to ensure optimal signal intensity and reproducibility.

### Calibration sample preparation for the novel LFA

To construct the calibration curve for the novel LFA, vancomycin-free serum samples were spiked with vancomycin at the following concentrations: 2.88, 14.40, 72, 360, 1,800, 9,000, and 45,000 ng/mL. Blood was collected from healthy adult volunteers and processed to obtain serum via centrifugation. Ethical approval for the study was obtained from the London Harrow Research Ethics Committee (19/LO/0219), and the study was registered on ClinicalTrials.gov (NCT04053140). Written informed consent was obtained from all participants in accordance with the Declaration of Helsinki. The methodology for serum preparation and spiking was adapted from validated clinical protocols for antibiotic quantification^23^.

### Calculation of LOD and LOQ for the novel vancomycin LFA

LOD and LOQ were calculated using the standard deviation (SD) of blank samples and the calibration curve obtained from the assay. Three blank (0 ng/mL) serum samples were measured to determine the mean blank signal and its standard deviation. The LOD was defined as the signal equal to the mean blank plus three times the SD, and the LOQ as the mean blank plus ten times the SD.

The calibration curve followed a power-law equation of the form: Y = 0.022 × x^0.6926^. Where y is the measured signal and x is the vancomycin concentration. To determine LOD and LOQ in concentration units (ng/mL), the corresponding signal values were substituted into the inverse of the calibration equation.

### Reproducibility and strip stability

The reproducibility of the novel vancomycin LFA was evaluated by assessing both intra-assay (within the same day) and inter-assay (between different days) precision. Three quality control concentrations—high (QC1: 45,000 ng/mL), medium (QC2: 360 ng/mL), and low (QC3: 2.88 ng/mL)—were tested. Each concentration was measured in five replicates (*n* = 5). Intra-day precision was assessed by running all replicates on the same day, while inter-day precision was evaluated by repeating the assay on five different days^24^.

To assess the stability of the novel LFA for vancomycin across different storage temperatures, a single batch of assembled LFA strips was divided into two sub-batches. One sub-batch was stored at room temperature, and the other at 4 °C for one week. After storage, all strips were tested using serum samples spiked with 2 µg/mL of vancomycin, and signal intensities were compared to freshly prepared strips.

### Specificity testing

To evaluate the specificity of the vancomycin LFA, we tested structurally related and unrelated antibiotics: teicoplanin (a glycopeptide) and ceftriaxone (a β-lactam antibiotic). Stock solutions of teicoplanin and ceftriaxone were prepared in serum and diluted to final concentrations of 10,000 ng/mL, 45,000 ng/mL, and 100,000 ng/mL. These concentrations were selected to cover typical therapeutic levels (10,000 ng/mL), the upper limit of the vancomycin LFA detection range (45,000 ng/mL), and a high excess level (100,000 ng/mL) to test worst-case cross-reactivity. Each condition was tested in triplicate. After the assay developed, test strips were measured using the same image analysis software used for vancomycin calibration.

The signal intensity produced by the interferents (teicoplanin and ceftriaxone) was converted into vancomycin-equivalent concentrations using the vancomycin calibration curve. The cross-reactivity was calculated using the formula: cross-reactivity (%) = (measured concentration of VAN) / (expected concentration of interferent) X 100^6^.

### Serum sample analysis and recovery

To evaluate the performance of the novel competitive LFA in a biologically relevant matrix, human serum samples were spiked with known concentrations of vancomycin and analysed using the developed assay. Three target concentrations were tested: 1,000 ng/mL, 500 ng/mL, and 70 ng/mL. Each concentration was tested in triplicate (*n* = 3). Recovery (%) was determined using the following formula:

Recovery (%) = (measured concentration) / (spiked concentration) X 100^23^.

## Results

### Characterisation of conjugates and receptor molecules

**Fig. 2:**
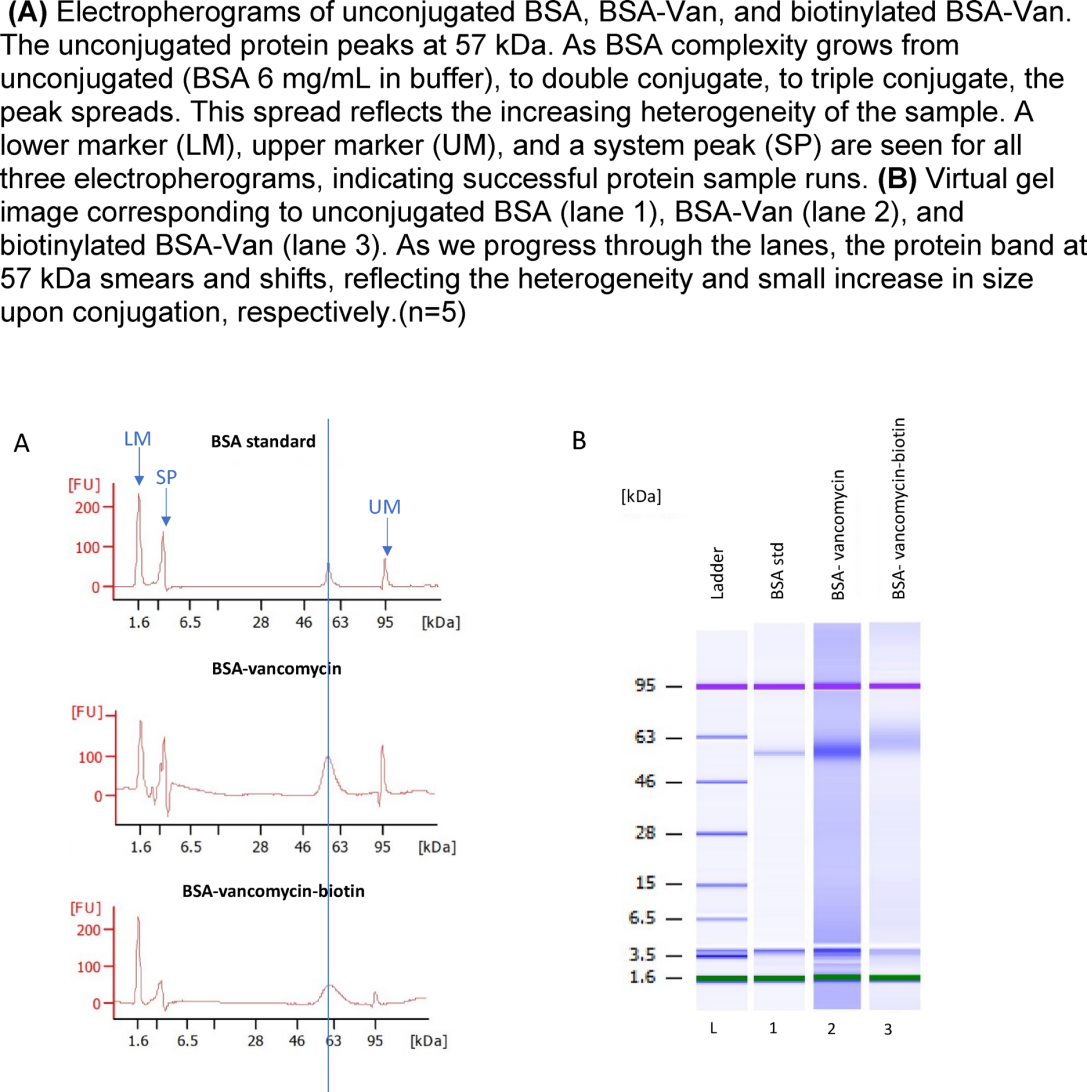
Protein 80 bioanalyzer results. (**A**) Electropherograms of unconjugated BSA, BSA-Van, and biotinylated BSA-Van. The unconjugated protein peaks at 57 kDa. As BSA complexity grows from unconjugated (BSA 6 mg/mL in buffer), to double conjugate, to triple conjugate, the peak spreads. This spread reflects the increasing heterogeneity of the sample. A lower marker (LM), upper marker (UM), and a system peak (SP) are seen for all three electropherograms, indicating successful protein sample runs. (**B**) Virtual gel image corresponding to unconjugated BSA (lane 1), BSA-Van (lane 2), and biotinylated BSA-Van (lane 3). As we progress through the lanes, the protein band at 57 kDa smears and shifts, reflecting the heterogeneity and small increase in size upon conjugation, respectively.(*n* = 5).

To confirm successful conjugation of vancomycin and biotin to BSA, the samples were analysed using the Protein 80 Bioanalyzer. As shown in Fig. 2, increasing sample heterogeneity was observed from unconjugated BSA to BSA–vancomycin (BSA–Van) and biotinylated BSA–Van. This broadening of peaks and band smearing reflects the progressive addition of chemical moieties and confirms successful conjugation.

### ELISA for vancomycin

**Fig. 3:**
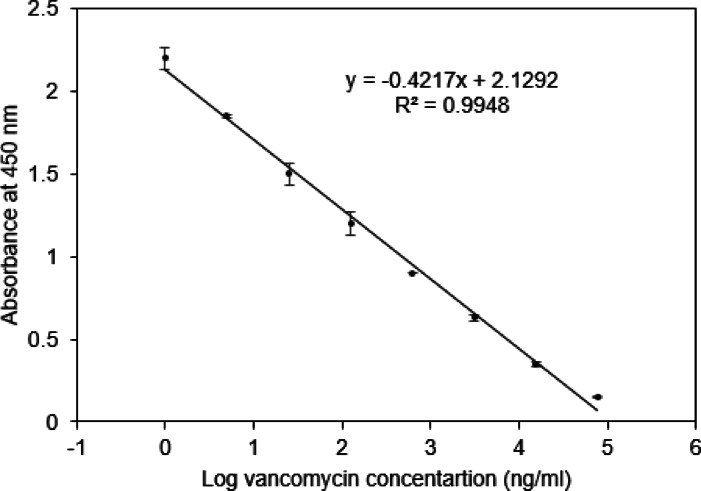
Calibration curve for the indirect competitive ELISA for vancomycin in serum samples (*n* = 3).

An indirect competitive ELISA was developed to evaluate the binding performance and sensitivity of the anti-vancomycin antibody prior to its integration into the lateral flow assay. The method demonstrated a sensitive detection range between 1 and 78,125 ng/mL of vancomycin, with a limit of detection (LOD) of 4 ng/mL and a limit of quantification (LOQ) of 12 ng/mL, as shown in (Fig. 3). Accuracy and precision were evaluated at three quality control concentrations (Qc1, Qc2, Qc3). Intra- and inter-day precision ranged from 4 to 8%, while accuracy ranged from 96 to 102% in serum samples (Table 1). These findings support the antibody’s suitability for use in the LFA platform and validate its potential for reliable vancomycin quantification.

**Table 1:** Inter- and intra-day variability, precision, and accuracy of the ELISA method for vancomycin.

	Spiking level ng/mL	RSD %	Accuracy %
Intra-day			
Qc1	1	4	102
Qc2	625	8	99
Qc3	78,125	7	101
Inter-day			
Qc1	1	5	101
Qc2	625	8	98
Qc3	78,125	6	96

### Traditional competitive LFA for Vancomycin

**Fig. 4:**
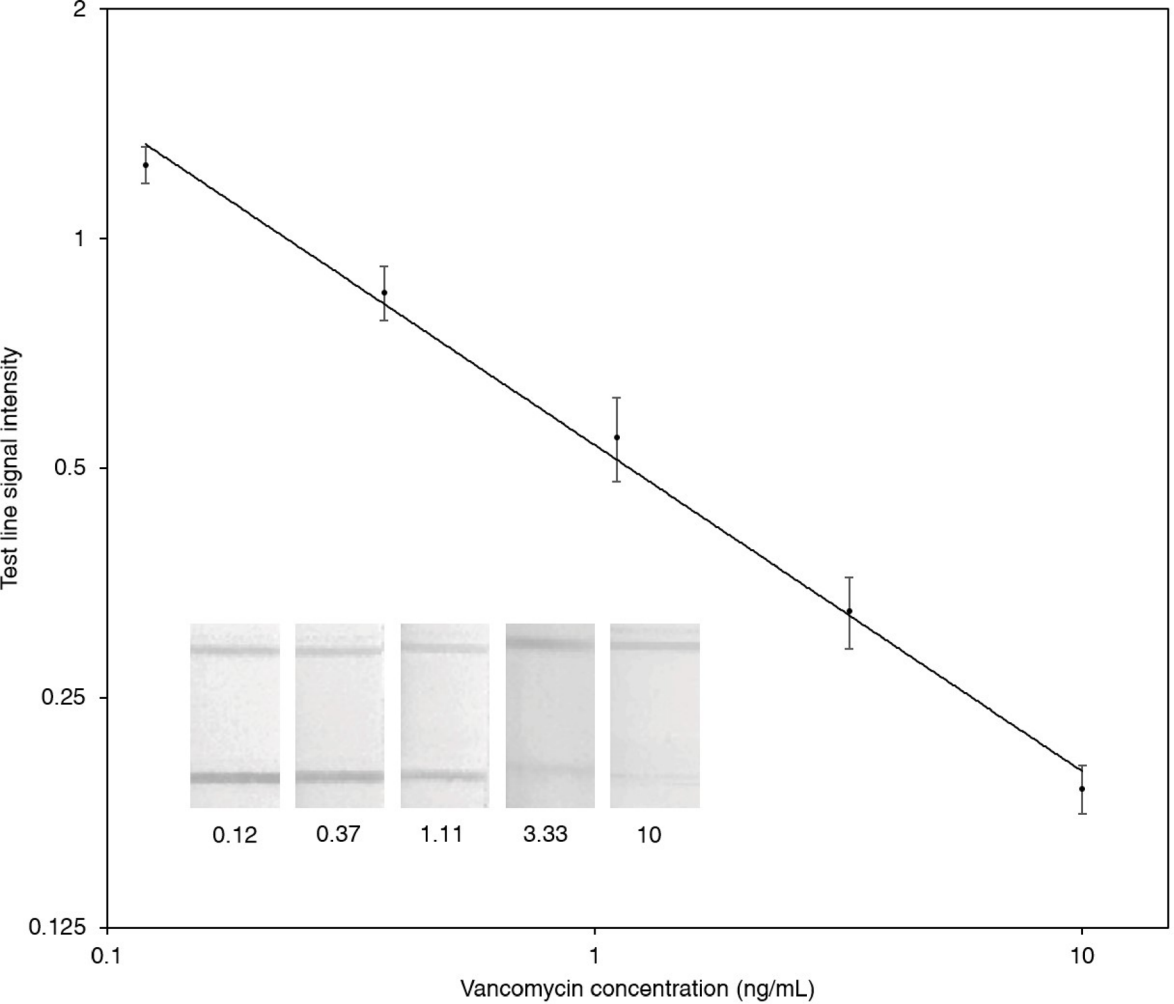
Calibration curve for the traditional competitive LFA for vancomycin with images of LFA strips with different concentrations of vancomycin. the number indicated vancomycin concentrations in serum samples, *n* = 3.

A dose-response curve for the traditional competitive LFA was generated using vancomycin concentrations ranging from 0.12 to 10 ng/mL spiked in blank serum, yielding a power-law fit with the equation y = 0.5369 × x^−0.428^ and an R^2^ value of 0.99, as shown in (Fig. 4).

### Novel competitive LFA for vancomycin

Image analysis of the novel LFA design indicated that the maximum intensity of each test line relative to the baseline, as obtained from the algorithm, provided more stable and consistent results compared to using the area under the curve of each test line. This approach demonstrated a stronger correlation with vancomycin concentration and reduced variability across repeated measurements.

**Fig. 5:**
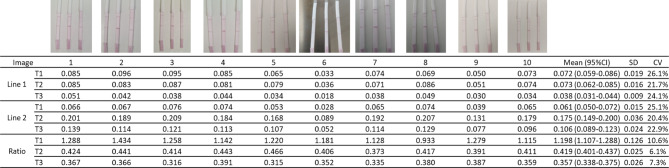
Accuracy testing of a set of three selected LFA strips with different camara model, light intensity, light source, and camera angle T1-T3: test strip from left to right of each picture, respectively, *CV* coefficient of variation, *SD* standard deviation.

Figure 5 illustrates the accuracy of three selected LFA strips, each subjected to varying camera models, light intensities, light sources, and camera angles. The adoption of the intensity proportion between the two lines yielded minimal variation, with a coefficient of variation (CV) below 10%, in contrast to the direct extraction of intensity from each test lines used in traditional competitive LFA, which exhibited a higher CV exceeding 20%.

**Fig. 6:**
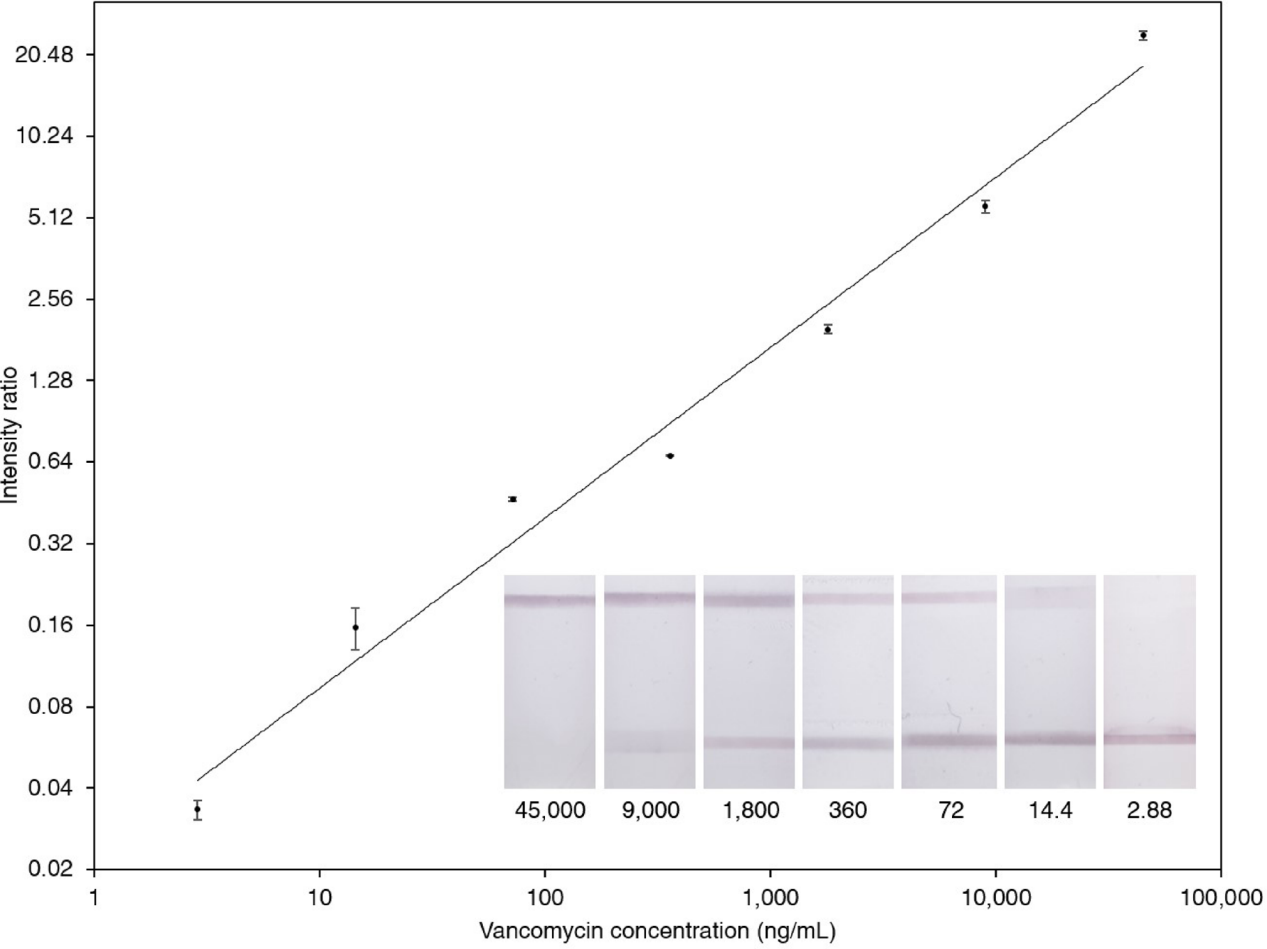
Calibration curve for the novel competitive LFA for vancomycin, alongside representative images of LFA strips tested with different vancomycin concentrations. The numbers indicate vancomycin concentrations in serum samples. Data represent mean values from triplicate measurements (*n* = 3).

Figure 6 shows the calibration curve obtained using this intensity ratio method, with vancomycin concentrations ranging from 2.88 to 45,000 ng/mL. The data followed a power-law relationship described by the equation: Y = 0.022 × x^0.6926^, where Y is the signal ratio and x is the vancomycin concentration (ng/mL). The fit was statistically significant (*p* < 0.0001) with a high adjusted R^2^ value of 0.9823, indicating excellent correlation between signal ratio and analyte concentration.

### Analytical sensitivity and blank sample response

**Fig. 7:**
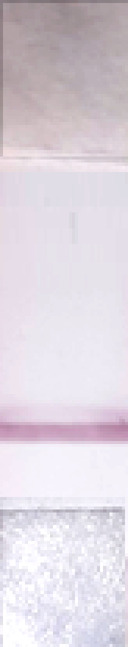
LFA strip for a blank serum sample (0 ng/mL vancomycin).

A blank serum sample (0 ng/mL vancomycin) was tested to assess novel competitive LFA performance in the absence of analyte. As shown in (Fig. 7), the LFA strip displayed a strong T1 line and no visible T2 line, consistent with the expected competitive binding mechanism and confirming specificity.

Based on three replicate blank measurements, the mean signal was 0.002 and the standard deviation was 0.0005. The calculated LOD and LOQ signal intensities were 0.0035 and 0.007, respectively.

Using the inverted calibration curve, these signals corresponded to approximately 1.94 ng/mL (LOD) and 4.87 ng/mL (LOQ) of vancomycin, demonstrating the high sensitivity of the assay.

### Reproducibility and storage condition

The reproducibility of the novel competitive LFA was evaluated based on intra-assay (within a single day) and inter-assay (across multiple days) precision. As shown in Table 2, the intra-assay and inter-assay relative standard deviations (RSDs) ranged from 5 to 11.5% and 5–12.5%, respectively. All RSD values were below 13%, indicating an acceptable level of precision for quantitative analysis using the vancomycin LFA strips.

**Table 2:** Inter- and intra-day variability and precision of the novel competitive LFA method for Vancomycin (*n* = 5).

	Spiking level ng/mL	RSD %
Intra-day		
Qc1	45,000	11.5
Qc2	360	8.5
Qc3	2.88	5
Inter-day		
Qc1	45,000	12.5
Qc2	360	8.9
Qc3	2.88	5

The intensity readings compared between day 1 and day 7 showed that LFA strips retained 95% of their signal when stored at 4 °C, whereas strips stored at room temperature (RT) retained only 60%, indicating reduced stability at RT.

### Specificity of the vancomycin LFA

The specificity of the vancomycin LFA was evaluated by testing potential cross-reactivity with teicoplanin and ceftriaxone at concentrations of 10,000, 45,000, and 100,000 ng/mL. The assay yielded vancomycin-equivalent signals of 23.15–55.16 ng/mL for teicoplanin and 18.76–49.89 ng/mL for ceftriaxone. These correspond to cross-reactivity percentages of ≤ 0.23% across all tested concentrations (Table 3). These results demonstrate the high specificity of the assay for vancomycin, with minimal interference from structurally related (teicoplanin) or unrelated (ceftriaxone) antibiotics.

**Table 3:** Specificity of the Vancomycin LFA against Teicoplanin and ceftriaxone, *n* = 3.

Interferent	Tested Conc. (ng/mL)	Measured vancomycin Eq. (ng/mL)	Cross-reactivity (%)
Teicoplanin	10,000	23.15	0.23
	45,000	27.12	0.06
	100,000	55.16	0.06
Ceftriaxone	10,000	18.76	0.19
	45,000	22.34	0.05
	100,000	49.89	0.05

### Serum sample analysis

To illustrate the potential application of the presented novel LFA in practical samples, blood samples spiked with different concentrations of vancomycin were tested using the novel competitive LFA as shown in (Table 4). The LFA demonstrated acceptable recovery values ranging from 82.8 to 117%, supporting its potential suitability for direct analysis of clinical serum samples.

**Table 4:** Testing novel competitive LFA using spiked blood samples, *n* = 3.

Spiked concentration ng/mL	Measured concentration by LFA ng/mL	Recovery %
1000	1170	117
500	506	101.2
70	58	82.8

## Discussion

The results of this study demonstrated that the traditional competitive LFA for vancomycin offers limited sensitivity compared to the novel competitive LFA developed in this work. This novel approach improved sensitivity for vancomycin TDM and demonstrates the feasibility of a dual-line competitive LFA format for quantitative drug monitoring. The single test line used in traditional competitive LFA, containing vancomycin IgG, produced lower signal at higher vancomycin concentrations, limiting its ability to accurately quantify across the range needed for effective TDM. The inherent design of a competitive assay leads to an inverse relationship between signal intensity and drug concentration, with a detection range constrained by the amount of conjugate displaced at the antibody binding site, whilst the intensity of the control line remains constant. Given that the clinically relevant trough concentration for vancomycin is 15–20 mg/L (15,000–20,000 ng/mL)^3^, the detection range of traditional competitive LFAs may be inadequate to support effective TDM for vancomycin. Although diluting samples 2,000–5,000 fold could theoretically bring vancomycin concentrations into the detectable range, such extreme dilution is impractical in routine clinical settings. Serial dilutions of this magnitude compound pipetting errors and introduce significant variability, compromising reliability—especially in point-of-care environments where specialized equipment and trained personnel are not available^25,26^.

Moreover, traditional LFAs that rely on a single test line are vulnerable to environmental variability. Factors such as camera type, lighting intensity, light source, and angle significantly affect signal intensity, as evidenced by accuracy testing, where coefficients of variation (CV) exceeded 20%. Addressing such variability typically requires specialised equipment—such as closed imaging boxes with fixed light sources—to ensure consistency^27–35^.

While some traditional LFAs have attempted to use signal ratios between the test and control lines for quantification, the control line in such formats typically functions only as a procedural check, with minimal variation in intensity across different analyte concentrations^36^. In contrast, our novel dual-line design introduces a second test line coated with avidin, which captures unbound biotinylated conjugates via high-affinity biotin–avidin interactions. This design results in a second signal that increases proportionally with vancomycin concentration. Unlike traditional control lines, which remain static and serve only as procedural checks, the second line in our system provides a dynamic, concentration-dependent response.

This configuration allows both test lines to contribute quantitatively, enabling ratio-based analysis that enhances sensitivity and robustness. The use of relative signal intensity between the two lines (rather than absolute intensity) further minimises the impact of external lighting or device variability. Because both lines and the membrane background are equally affected by illumination, the ratio remains consistent under variable ambient conditions—unless there are localised artefacts such as operator shadows.

By combining this innovative dual-line layout with a custom R-based image analysis algorithm, the assay achieves improved detection range, precision, and environmental stability. These features make the LFA particularly suitable for serum antibiotic monitoring across diverse healthcare environments. Its simplicity and fast performance support use in low-resource settings, while smartphone-based image capture enables practical bedside TDM in advanced care settings.

The calculated limit of detection (LOD) and limit of quantification (LOQ) for the assay were 1.94 ng/mL and 4.87 ng/mL, respectively, based on the standard deviation of the blank and the fitted calibration curve. These values indicate the assay’s strong analytical sensitivity and support its potential use for quantifying vancomycin at low concentrations. While the LOD is lower than the lowest concentration used in the calibration curve (2.88 ng/mL), this is consistent with how LOD is defined statistically—as the minimum concentration that can be distinguished from background noise. In contrast, the calibration range reflects concentrations that were experimentally validated. Therefore, although 2.88 ng/mL represents the practical starting point of the assay’s quantifiable range, the lower LOD highlights the sensitivity and robustness of the platform.

Building on these promising results, future work could focus on further validation using clinical samples and collaboration with external laboratories to confirm reproducibility. Additionally, while the current dual-test-line design eliminates the need for a traditional control line, future iterations of the assay could explore the incorporation of a dedicated control line using an alternative nanoparticle to support internal validation of proper sample flow. Importantly, the current study relied on spiked serum rather than actual patient samples, and no comparison was made with gold-standard reference methods such as LC-MS/MS. The assay’s long-term stability, performance under field conditions, and batch-to-batch consistency also require further investigation. Addressing these factors will be essential to translate this prototype into a robust, clinically deployable tool.

In conclusion, we developed a novel, user-friendly lateral flow assay (LFA) for the quantitative detection of vancomycin in serum. The assay utilises a competitive two-test-line immunoassay format, designed to improve both sensitivity and robustness against environmental variability. It delivers rapid results suitable for near-patient use. Analytical validation demonstrated good sensitivity, specificity, reproducibility, and recovery, suggesting its potential for future clinical integration. While further validation with patient samples is required, this LFA offers a promising platform to support timely and decentralised TDM of vancomycin, which could contribute to improved dosing precision and antimicrobial stewardship. Ongoing optimisation and clinical benchmarking will be key to realising the full translational potential of this assay.

## Electronic supplementary material

Below is the link to the electronic supplementary material.

Supplementary Material 1

## Data Availability

The R code written for LFA reading is available at: https://github.com/vvasikasin/LFA_CAMO. The datasets used and/or analysed during the current study available from the corresponding author on reasonable request.
