# Supplementary material for: Development of a novel quantitative lateral flow assay for vancomycin for therapeutic drug monitoring
Source: Sci Rep. 2025 Jul 8;15:24398. doi: 10.1038/s41598-025-09145-1 (PMC12238248; doi:10.1038/s41598-025-09145-1)
Supplement: Supplementary file 1 — Supplementary Material 1 [file 41598_2025_9145_MOESM1_ESM.docx]

# Supplementary Appendix 1

# Image processing algorithm

## Colour adjustment

The initial step involved manual cropping of the image, followed by conversion into grayscale using the mean of three-color intensities (red, green, and blue; RGB). The choice of grayscale instead of reliance solely on the red colour of the AuNP was made for several reasons. Firstly, potential colour fluctuations during development, attributed to imperfect protein-AuNP conjugation leading to aggregation, could result in lower wavelengths and a colour shift toward purple, such as appearing bluer instead of red. Secondly, variations in smartphone algorithms for white balance and RGB could affect colour representation. Utilising the mean of each RGB colour mitigated differences across smartphone models. Lastly, grayscale was proven effective in a previous study assessing LFA for digoxigenin quantification (1).

The mean intensity values of each pixel obtained from the grayscale conversion were transformed into a two-dimensional dataframe. Each row represented horizontal pixels, and each column represented vertical pixels. Adhering to the guidance from the nitrocellulose membrane manufacturer (Millipore, Darmstadt, Germany), which specified a linear flow front for the sample on the membrane, it was assumed that the intensity of each row should be relatively uniform. Figure S1 illustrates demonstrates the process.

Figure S1 The process of cropping manually and converting into a two-dimension dataframe containing the intensity values obtained from the mean value of each colour in RGB.


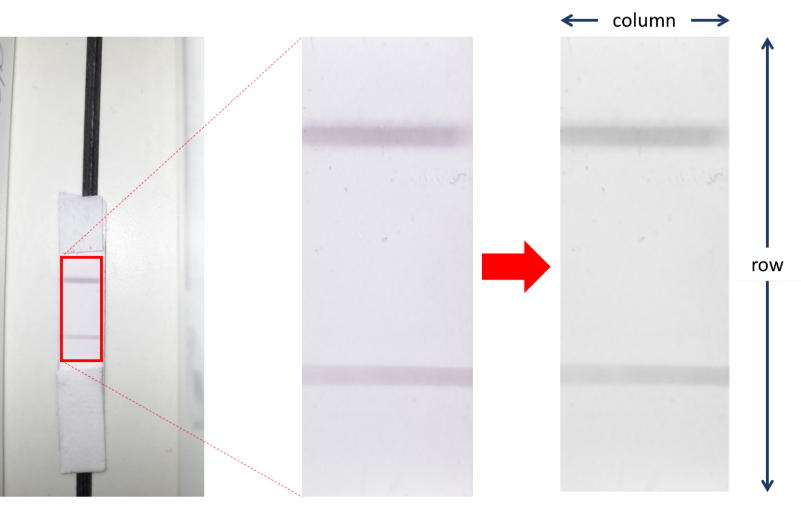


## Artefact removal

Upon initial image analysis, various artefacts were identified within the images. Small ambient dust particles could adhere to the nitrocellulose membrane surface, and irregularities in the test lines might arise from imperfections in strip printing. To mitigate these artefacts, a decision was made to exclude rows with extremely high or low intensities. Specifically, intensities exceeding the 95th percentile or falling below the 5th percentile for each row were excluded from the analysis. This process is illustrated in Figure S2, demonstrating the effective removal of undesired elements to enhance the reliability of subsequent analyses.

Figure S2 Examples of how the artefacts were removed The green circles show the area of the second test line which has lower intensity than others in the same rows due to the error in printing. The red circles show a dust particle that can increase the intensity. Both can be removed by the algorithm. Black represents the NA values not included in the further analysis.


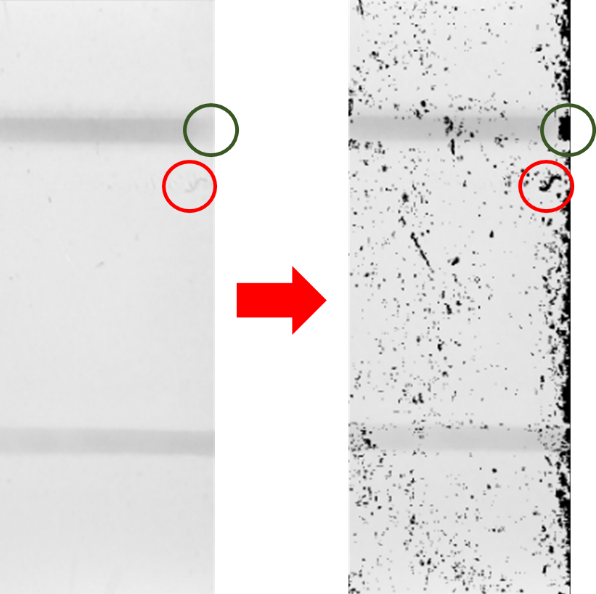


## Baseline reduction and signal denoising

Subsequently, the mean intensity of each row, as plotted in Figure S3(a), was calculated. An additional challenge observed during the initial analysis was the linear intensity decrease in the baseline background, with the first rows closer to the conjugated pad exhibiting a different trend compared to the last rows closer to the absorbent pad, as illustrated in Figure S3(a). This phenomenon could arise from variations in the diffusion of AuNP on the nitrocellulose membrane or differences in camera angle and lighting conditions.

To adjust for this difference, the median intensity of the first, middle, and last 20 rows was utilised as three fixed points, assuming they should ideally possess identical values. This approach was based on the assumption that no test line is positioned in the middle. This adjustment involved subtracting the mean of each row with the slope values between these three points, as shown in Figure S3(b). This corrective measure aimed to standardise the baseline intensities, minimising the impact of variations induced by AuNP diffusion and imaging conditions on subsequent analyses.

Figure S3 The plot obtained from the mean intensity of each row (a) The fixed three points, represented with the red circles, that were used to adjust the baseline. (b) The result after baseline adjustments.


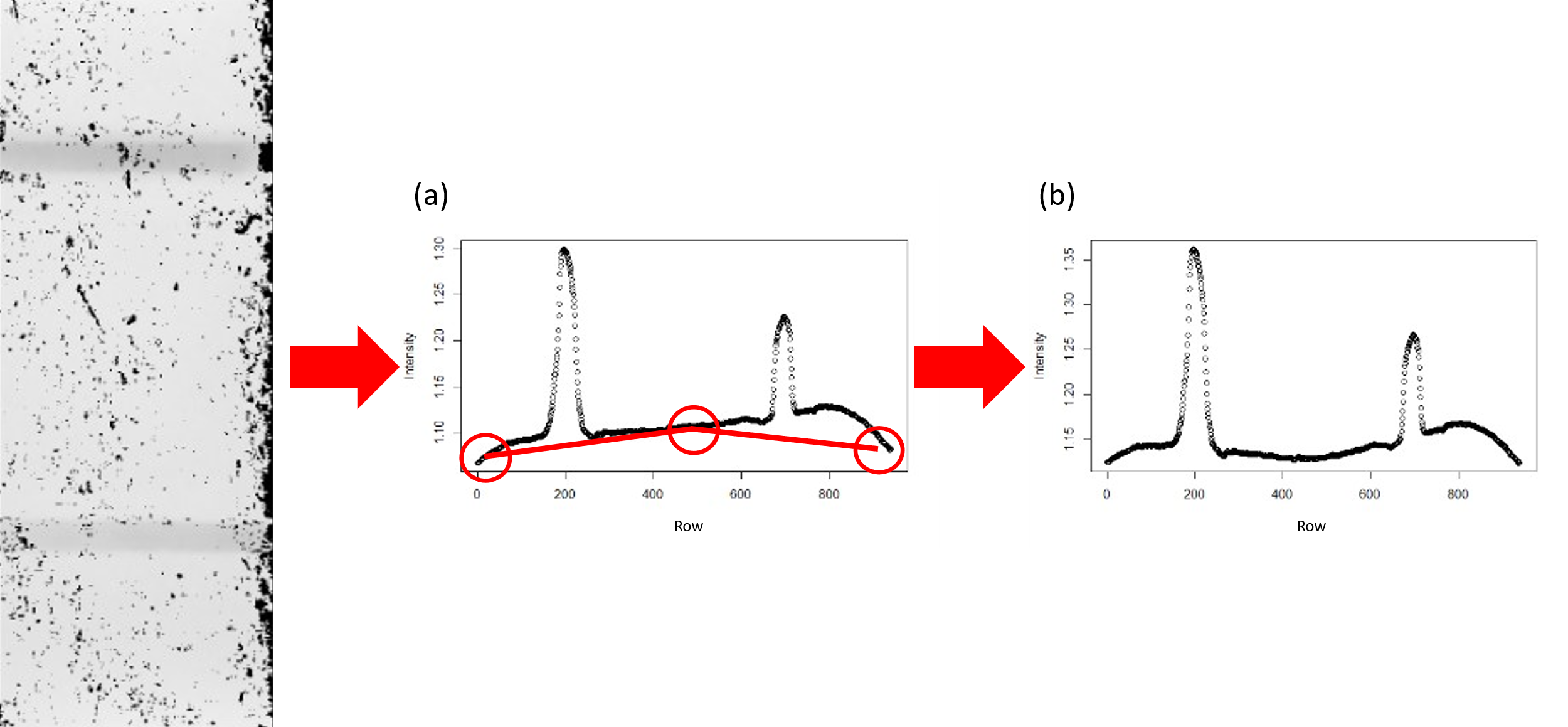


To further diminish noise, a moving average was applied to each row using the adjacent 10 rows, as shown in Figure S4(a). Despite this, the baseline exhibited non-uniform flatness, necessitating additional refinement.

In addressing this challenge, similar to issues encountered in various forms of spectroscopy, baseline adjustments were implemented. In the field of spectroscopy, baseline correction has been a recurrent concern, with the primary goal being to identify the baseline type, estimate it, and subtract it from the overall signal (2). Various sophisticated approaches such as polynomial fitting, Fourier transform, penalised least squares, asymmetric least squares (ALS), Tikhonov regularisation, among others, have been employed for this purpose (2-4). To simplify this process, an R package "baseline" was used, providing multiple approaches that can be applied individually or simultaneously to LFA.

Asymmetric Least Squares (ALS) method was chosen for its efficacy in achieving a flat baseline with consistent results upon testing with different images (5). The parameters used included a second derivative constraint (lambda) of 5, weighting of positive residuals (p) at 0.01, and a maximum number of iterations (maxit) set to 20. The outcome after adjustment is presented in Figure S4(b). Consequently, the values of each row underwent a transformation from crude intensity to relative intensity when compared to the adjusted baseline.

Figure S4 The moving average and the baseline adjustment performed, (a) and (b), respectively.


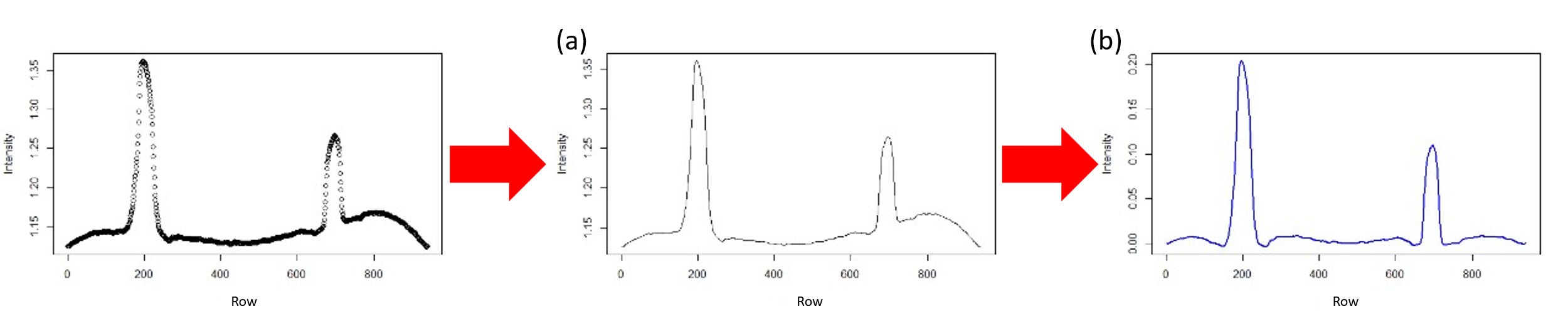


## Test line identification

For the automated identification of the two test lines, the following algorithm was employed. Initially, the median of all rows was calculated. Subsequently, the rows were divided into two zones: the first zone and the second zone. This division was based on the assumption that the first and second test lines are positioned on either side of the image, with no test line positioned in the middle. The test lines were then identified as the largest areas in each zone, defined as the longest consecutive rows where the intensity surpasses the median of all rows.

## Values extraction

Various parameters were extracted from the algorithm to determine the equation with the highest accuracy. These parameters included the area under the curve (AUC) and the maximum intensity of each test line. These parameters were fitted on calibration curves against the concentrations with different types of curve fitting, including linear regression (y=ax+b) and non-linear regression, such as quadratic fit (y=ax^2^+bx+c), logarithmic fit (y=aLn(x)+b), and power-law relationship (y=ax^b^). Coefficient of determination (R^2^) was used to determine the most suitable parameters and curve fitting methods in quantifying antibiotic concentrations.

# References

1. Ruppert C, Phogat N, Laufer S, Kohl M, Deigner HP. A smartphone readout system for gold nanoparticle-based lateral flow assays: application to monitoring of digoxigenin. Mikrochim Acta. 2019;186(2):119.

2. Mishra P, Biancolillo A, Roger JM, Marini F, Rutledge DN. New data preprocessing trends based on ensemble of multiple preprocessing techniques. TrAC Trends in Analytical Chemistry. 2020;132:116045.

3. Roger J-M, Boulet J-C, Zeaiter M, Rutledge DN. 3.01 - Pre-processing Methods☆. In: Brown S, Tauler R, Walczak B, editors. Comprehensive Chemometrics (Second Edition). Oxford: Elsevier; 2020. p. 1-75.

4. Ye J, Tian Z, Wei H, Li Y. Baseline correction method based on improved asymmetrically reweighted penalized least squares for the Raman spectrum. Appl Opt. 2020;59(34):10933-43.

5. Boelens HF, Dijkstra RJ, Eilers PH, Fitzpatrick F, Westerhuis JA. New background correction method for liquid chromatography with diode array detection, infrared spectroscopic detection and Raman spectroscopic detection. J Chromatogr A. 2004;1057(1-2):21-30.
